# Noncommunicable Diseases and Injuries in Latin America and the Caribbean: Time for Action

**DOI:** 10.1371/journal.pmed.0030344

**Published:** 2006-09-05

**Authors:** Pablo Perel, Juan P Casas, Zulma Ortiz, J. Jaime Miranda

## Abstract

The region is in a privileged position to quickly translate investment in health research into practice, argue Perel and colleagues.

The great burden that noncommunicable diseases (NCDs) and injuries have on low-income and middle-income countries is well recognized [1,2]. Latin American and Caribbean (LAC) countries as a group of middle-income countries are no exception to this neglected epidemic. In this Essay, we review the impact on public health of NCDs and injuries in LAC countries, as well as describe the regional particularities behind this epidemic. We discuss the reasons why LAC countries are in a privileged position to quickly translate investment in health research into practice. Finally, we describe possible research needs and the implications of this research for clinical practice and health policy in the region.

## Epidemiological Profile of LAC Countries

**Figure 1:**
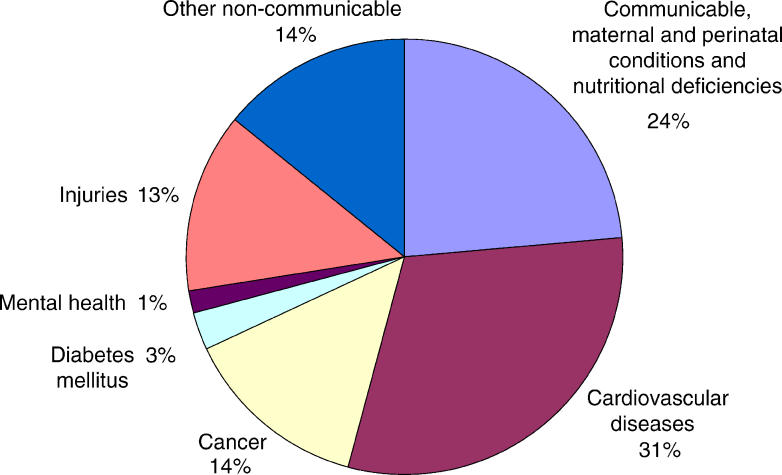
Distribution of Total Deaths (3,537,000) by Major Causes in LAC Countries in 2000, Estimated by the Global Burden of Disease Study The Latin American and Caribbean countries included are Anguilla, Antigua and Bermuda, Argentina, Aruba, Bahamas, Barbados, Belize, Bolivia, Brazil, British Virgin Islands, Cayman Islands, Chile, Colombia, Costa Rica, Cuba, Dominica, Dominican Republic, Ecuador, El Salvador, French Guiana, Grenada, Guadalupe, Guatemala, Guyana, Haiti, Honduras, Jamaica, Martinique, Mexico, Montserrat, Netherlands Antilles, Nicaragua, Panama, Paraguay, Peru, Puerto Rico, Saint Kitts and Nevis, Saint Lucia, Saint Vincent and the Grenadines, Suriname, Trinidad and Tobago, Turks and Caicos Islands, Uruguay, US Virgin Islands, and Venezuela.

**Figure 2:**
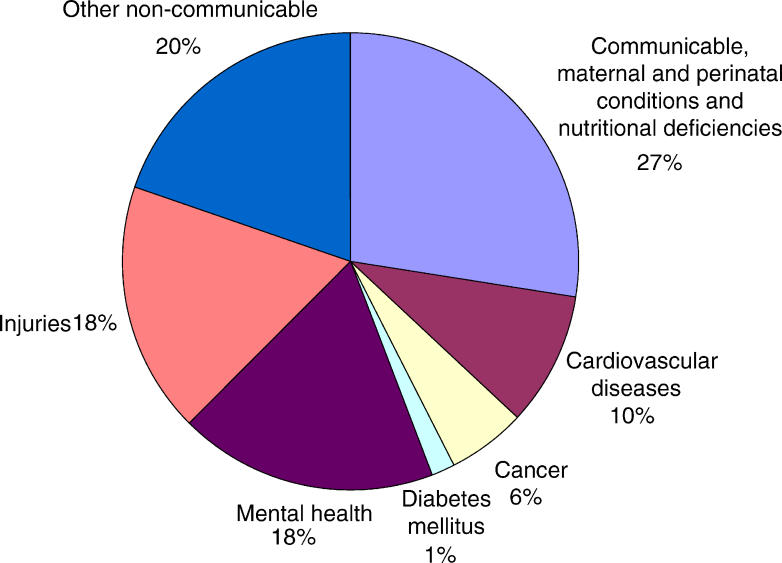
Distribution of Total DALYs (102,108,000) by Major Causes in LAC Countries in the Year 2000, Estimated by the Global Burden of Disease Study Latin American and Caribbean countries included are listed in Figure 1.

LAC countries, with a combined population of about 533 million people, have been experiencing in the last decades a rapid, complex epidemiological transition. By 1990, NCDs and injuries had already accounted for 69% of deaths and 65% of disability-adjusted life years (DALYs), a pattern still evident in 2000 (73% of deaths and 76% of DALYs). The largest impact on mortality was a result of cardiovascular disease, while for DALYs the greatest proportion was due to two neglected health problems: mental illness and injuries (Figures 1 and 2) [3]. This dominance of NCDs and injuries over infectious disease is expected to rise significantly by 2020, when the ratio of deaths from NCDs and injuries to deaths from infectious disease might increase from 2.2 to 8.1; likewise, a similar increase is expected to occur with the ratio of DALYs, increasing from 1.8 to 6.9 [3].

## The Epidemic of NCDs and Injuries in LAC Countries

In addition to urbanization, the two major driving forces of this epidemiological transition are globalization and the aging of the population. Life expectancy in LAC countries increased from 63.4 years in the period 1975–1980 to 72.2 in the period 2000–2005, and the population aged 65 years and older is expected to almost double, from 5.5% in 2000 to 9.8% in 2025 [4]. Globalization, including the globalization of the mass media, has contributed to an increase in unhealthy lifestyles in LAC countries, such as worsening dietary patterns and lower levels of physical activity. These changes in lifestyle have in turn contributed to the epidemic of NCDs and injuries [5]. In addition, there are a number of features that are particular to the region that have also played a role in the rise in NCDs and injuries.

## Social Inequality

The LAC region has the highest level of social inequality of any region in the world. In 1990 the wealthiest 20% of the LAC population earned 52.4% of the area’s income, while the poorest 20% earned only 4.5% [6]. This inequality is not only greater than that in developed countries but also greater than the inequality observed in other developing countries, as evaluated by the GINI coefficient—a measure of income inequality. In 1990 the GINI coefficient was 0.51 for LAC countries, a value higher than that for Africa (0.46), the Middle East (0.38), Southeast Asia, (0.37), or Eastern Europe (0.29) [6,7].

Social inequality is reflected in inequities in access to health care. The poor have worse access to care, which means they have less opportunity to receive treatment to control risk factors for chronic disease (e.g., high blood pressure) and to manage established chronic diseases and injuries [8]. And, as in developed countries, social inequalities are also reflected in differences in health status and risk factors independent of access to health care [9,10]. In Peru, a recent study showed that individuals with the lowest socioeconomic status had a 4-fold increased risk of having multiple cardiovascular risk factors compared to those with the highest socioeconomic status [9].

Another important and often neglected effect of social inequality is its impact on mental health and injuries in the young. Individuals from deprived areas, with lower educational levels, have the highest prevalence of mental health problems, as recently described in Colombia [11]. The LAC region has one of the world’s highest rates of intentional injuries (including homicide)—more than twice the world average—affecting disproportionably young men from disadvantaged social groups [3,12]. The LAC region also has a high rate of unintentional injuries, such as road traffic crashes, which are strongly and positively associated with poverty levels and lower literacy rates [13].

In addition to the effects of social inequalities on the epidemic of NCDs and injuries, the epidemic also contributes to the impoverishment of families and places an economic burden on the already-limited health-care budgets of LAC countries. This economic burden is accounted for not only by the high incidence of NCDs and injuries, but also by the high probability of developing disease at younger ages and often presenting with a poorer prognosis [14].

### Urbanization and forced rural–urban migration

In the LAC region, the proportion of urban population increased from 56.5% in 1970 to almost 78% in 2005 [4]. Associated with this unplanned urbanization in LAC countries is the growth of poor, peripheral urban settlements and a rapid rise in the number of middle-sized cites, added to the already-existing “mega-cities” of Latin America [15]. Furthermore, in the last two decades in some LAC countries, this urbanization has been fueled by a second wave of rural–urban migration—usually as a consequence of internal civil violence.

In Colombia, it is estimated that in the last 15 years around 2 million people (4.3% of the total population) have been forcibly displaced from their rural communities as a result of armed conflict [16]. Importantly, the low educational and socioeconomic level of this new wave of forced migrants has led them to settle in the existing poor, peripheral urban areas [16]. A similar scenario has occurred over the past 20 years in Peru, where the population of Lima increased from 3.5 million people in 1972 to 8 million in 2002—a phenomenon that was mainly caused by social violence [17]. Although there is no clear evidence of the consequences of this forced migration on NCDs and injuries, it is likely that this migration further increases the existing social inequalities. In turn, worsening inequalities affect individual and public health.

### Lack of adequate local and regional funds

In 2003 funds allocated to research and development (R&D) in the LAC region accounted for only 0.57% of the gross domestic product (http://www.ricyt.edu.ar), which lags behind other countries such as the United Kingdom (1.9%) or the United States (2.58%) (Chapter 4 of [18]). These scarce funds for R&D are also reflected in the poor output of research publications. In 1996, for example, only 2.1 Latin American articles were registered by the Institute for Scientific Information for each million dollars allocated for R&D; in Spain, this same quantity of resources generated four Institute for Scientific Information-registered articles [19].

Moreover, the limited funds for R&D in the LAC region are not usually allocated in proportion to the current burden of disease. A review of scientific production, an accepted proxy of research conducted, showed that in the LAC region a large majority (83%) of epidemiological publications focused on infectious diseases [20].

## Translating Investment in Health Research into Practice

In the LAC region, rapid translation of research investment into improved public health is highly feasible [21]. As a group of middle-income countries, the LAC countries have an adequate technology-communications infrastructure, as well as highly qualified health and academic human resources—essential for conducting high-quality human epidemiological research. Furthermore, LAC countries share a similar cultural identity, and most of the population shares a common language, which undoubtedly would strengthen regional initiatives in collaborative research. These favorable advantages, added to the low cost of conducting research in the region compared with the costs in developed countries, have been used by pharmaceutical companies to actively recruit LAC populations into randomized clinical trials. The number of US-based trials executed in LAC countries has increased over 10-fold from 1995 to 2000, making the LAC countries the fourth-largest clinical trial market [22]. Unfortunately, these clinical trials often do not address diseases with a great burden to the region (e.g., mental health, chronic respiratory disease, and trauma). In addition, trials conducted in the LAC population, while sometimes addressing relevant pathologies such as cardiovascular disease, usually involve interventions that would not be affordable to LAC populations or that would be difficult to implement in settings with inadequate health-care resources [22]. Nevertheless, in recent years examples of relevant trials conducted in LAC countries using affordable interventions are emerging, such as the CRASH, DIAL, and BENEFIT trials (Box 1) [23–25]. There are also examples of observational epidemiology evaluating questions relevant to the LAC region, such as the PLATINO [26] and INTERHEART [27] studies (Box 1).

**Box 1. Studies Relevant to LAC Countries**

Trials conducted in LAC countries using affordable interventions:

- **BENEFIT trial:** “The purpose of this study is to determine if 60 days of treatment with an antiparasitic drug (benznidazole) could prevent the progression of cardiac disease in patients with Chagas disease” (http://clinicaltrials.gov/ct/show/NCT00123916).
- **CRASH trial:** “A large simple, placebo controlled trial of the effects of a 48-hour infusion of corticosteroids on death and on neurological disability, among adults with head injury and some impairment of consciousness” (http://www.crash.lshtm.ac.uk/TP_English_StudyDesgn.htm).
- **DIAL trial:** A randomized trial “to see whether a program based on centralized telephone intervention performed by trained nurses can reduce the high rates of morbidity and mortality associated with chronic heart failure compared with ‘usual care’ administered by an attending cardiologist” (http://www.medscape.com/viewarticle/444970).

Observational studies evaluating questions relevant to LAC countries:

- **INTERHEART study:** “The aim of INTERHEART, a case-control study conducted in > 50 countries, was to determine the associations between a wide array of risk factors and AMI [acute myocardial infarction] within populations defined by ethnicity and/or geographic region, and to assess the relative importance of these risk factors across these populations” (http://www.medscape.com/viewarticle/489738).
- **PLATINO study:** The aim of this study, launched in 2002, “was to describe the epidemiology of COPD [chronic obstructive pulmonary disease] in five major Latin American cities: São Paulo (Brazil), Santiago (Chile), Mexico City (Mexico), Montevideo (Uruguay), and Caracas (Venezuela)” [26].

## Why LAC Countries Need More Research on NCDs and Injuries

Some would argue that there is no need for regional research since studies conducted anywhere in the world should be applicable to different settings. Although we consider that most of the biological determinants of NCDs and injuries in LAC are likely to be similar to those in other parts of the world, the population-attributable risk and the circumstances in which those disease determinants arise and progress are indeed different between populations. Understanding the impact of rural–urban migration (unforced and forced) and urbanization on risk factors for NCDs and injuries is crucial. The effects of the existing, large social inequalities on the development and progression of NCDs and injuries and their risk factors may differ within and between LAC countries, and these effects may be different within the LAC region compared with the rest of the world [28].

Evidence derived from studies conducted in developed countries has suggested that the rise in chronic diseases is largely explained by a few modifiable risk factors (smoking, blood pressure, cholesterol, and obesity), and that interventions aimed at producing small changes in those risk factors at population level are highly cost-effective [1]. However, the development and implementation of such policies in developing countries is a complex challenge.

Regional research is therefore of great importance since some interventions, such as changing behavior, for preventing NCDs and injuries with long, complex pathways could be effective in one setting but ineffective in a resource-poor context—a phenomenon known as “transferability” of evidence [29]. For example, a systematic review of street lighting showed a protective effect on road traffic crashes, but all of the data came from developed countries [30]. To assume that this intervention would be just as effective in LAC countries is inappropriate because of behavioral, vehicular, and environmental differences [30].

In addition, some health problems are of greater relevance to LAC countries (e.g., cardiomiopathy due to Chagas disease and road traffic injuries) than to developed countries, and randomized evidence to support effective interventions to address these problems is urgently needed [31,32]. Finally, as previously mentioned, many treatments that are affordable in developed countries are beyond the reach of a large proportion of the LAC population.

There is one additional benefit that would be associated with a growth in regional research in LAC countries. If more health practitioners were to get involved in such research, they would become familiar with the principles of research methodology, and this in turn may stimulate their practice of evidence-based medicine. Greater use of the best-available evidence may be beneficial to population health.

Although we believe that the global burden of disease should be used to guide research priorities in the LAC region, we acknowledge that there are additional considerations. First, different LAC countries are at different stages of the epidemiological transition, and so each country needs its own specific research agenda. Second, this agenda should also consider issues such as addressing health inequality and improving health-care services. Third, the process of establishing the research agenda must include all the relevant sectors of the society (government, the public, academia, nongovernmental organizations, and the private sector) [33,34].

## Abbreviations

DALY: disability-adjusted life year
LAC: Latin American and Caribbean
NCD: noncommunicable disease
R&D: research and development
